# Stress-field driven conformal lattice design using circle packing algorithm

**DOI:** 10.1016/j.heliyon.2023.e14448

**Published:** 2023-03-13

**Authors:** Fuyuan Liu, Min Chen, Lizhe Wang, Tianheng Luo, Geng Chen

**Affiliations:** a School of Advanced Technology, Xi'an Jiaotong – Liverpool, University, Suzhou, 215000, Jiangsu, China; b School of Mechanical, Electronic and Control Engineering, Beijing Jiaotong University, Beijing, 100000, Beijing, China

**Keywords:** Organic Strut-based lattice, Stress-field driven, Conformal design, Circle packing algorithm, Genetic algorithm

## Abstract

Reliable extreme lightweight is the pursuit in many high-end manufacturing areas. Aided by additive manufacturing (AM), lattice material has become a promising candidate for lightweight optimization. Configuration of lattice units at the material level and the distribution of lattice units at the structure level are the two main research directions recently. This paper proposes a generative strategy for lattice infilling optimization using organic strut-based lattices. A sphere packing algorithm driven by von Mises stress fields determines the lattice distribution density. Two typical configurations, Voronoi polygons and Delaunay triangles, are adopted to constitute the frames, respectively. Based on finite element analysis, a simplified truss model is utilized to evaluate the lattice distribution in terms of mechanical properties. Optimization parameters, including node number, mapping gradient, and the range of varying circle size, are investigated through the genetic algorithm (GA). Multiple feasible solutions are obtained for further solidification modelling. To avoid the stress concentration, the organic strut-based lattice units are created by the iso-surface modelling method. The effectiveness of the proposed generative approach is illustrated through a classical 3-point bending beam. The stiffness of the optimized structure, verified through experimental testing, has increased 80% over the one using the traditional uniform body center cubic (BCC) lattice distribution.

## Graphical abstract

**Figure:**
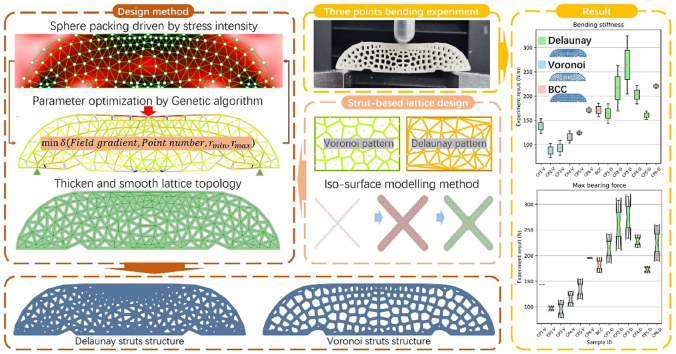

## 1 Introduction

Lightweight design for high specific stiffness is a pursuit for high-end industrial devices, like E-vehicles, industrial robots, and UAVs [1]. The main reason is that lightweight design saves material and reaches comprehensive improvements in durability, mobility, and energy consumption. With the development of additive manufacturing (AM), lattice material has become popular for enhancing AM-based lightweight components. Especially its low density and customized structure bring superb mechanical properties, i.e., high specific stiffness [2], energy absorption [3], and thermal insulation [4], benefiting optimizing functional parts. A challenge is how to design and infill lattice materials for specific stiffness (stiffness-to-weight ratio) improvement.

**Fig. 1:**
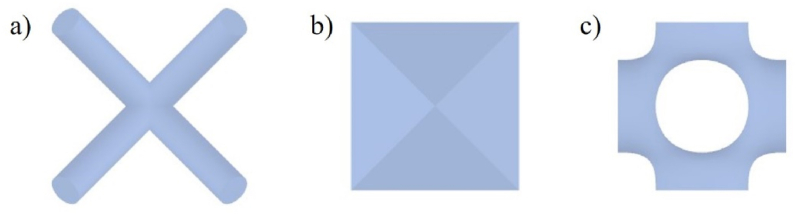
Different types of lattices a) Struts-based lattice b) Sheet-based lattice c) TPMS lattice.

The lattice morphology on both material and structure levels greatly influences the mechanical performance of components. On the material level, lattice material is a periodic cluster of structural units. The mesoscale units determine the mechanical property of lattice material [5]. Lattice units can be divided into two main classes, geometric ones, such as struts-based lattice and plate-based lattice, and organic ones, like triple periodic minimum surface (TPMS) lattice, according to its morphology, as seen in Fig. 1(a–c). As increasing the degree of design complexity, strut-based lattice often adapts to design and formulate a conformal structure due to its simple and easy-editable topologies [6]. Scholars made much effort in the configuration design to improve the mechanical properties of struts-based lattices. For example, Nguyen et al. regulated and optimized the position and orientation of the struts to improve the stiffness of the lattice under six loading conditions (XX, YY, ZZ, YZ, XY, XZ) [7]. Xu et al. proposed two design strategies regulating the anisotropy of the struts-based lattice by assembling different base units and adjusting the ratio of rod diameters, respectively [8]. In addition, Maxwell's principle provides clear guidance for designing high-stiffness strut-based lattices [9]. The principle defines the mechanical behavior of lattice material, stretch-dominated to high stiffness design or bending-dominated to energy absorption, by the number of nodes and rods. However, a stress concentration at nodes still limits the strength of lattice material [10]. A solution is to make strut-based lattice struts organic and smooth, like a streamlined surface of TPMS lattice. Recent studies demonstrated that organic lattice morphology can greatly improve mechanical performance. For instance, Wang et al. filleted the joints of gradient lattice structures to increment the stiffness [11]. Lee et al. utilized the Bezier curve to construct the profile of lattice struts and curve parameters with deep learning and genetic algorithm, which remarkably improved the elastic modulus of strut-based lattice [12]. Making a high-strength lattice configuration organic can further develop the mechanical properties of lattice material.

**Fig. 2:**
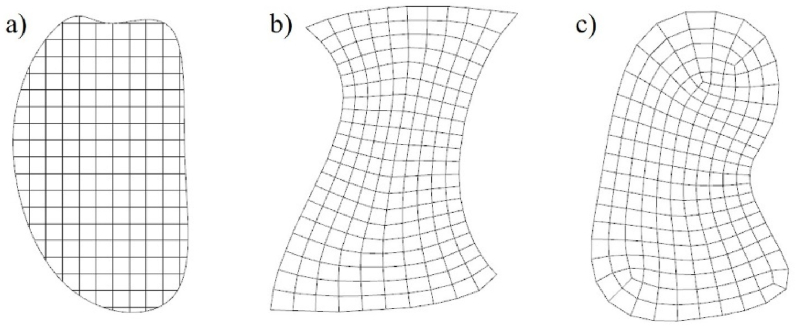
Three infilling methods: a) Trimming b) Sweeping c) Meshing.

On the structure level, conformal design and high specific stiffness are two design factors for infilling optimization. The conformal design maintains the structural integrity; specific stiffness enhancement improves the functional parts [13]. However, traditional lattice infilling methods, such as meshing (Fig. 2 (a)), sweeping (Fig. 2 (b)), and trimming (Fig. 2 (c)), fail to consider the influence of external loads [14].

Infilling gradient lattice materials (GLMs) can make up for the shortcomings of the above methods [15]. In present studies, two strategies, including stress field-driven design or topology optimization, can construct high-stiffness lattice structures under the influence of external loads. Nguyen et al. infilled customized AM-lattice based on density-variable topology optimization [16]. Arora et al. generated a Michell truss guided by the stress tensor, which gained an editable volumetric lattice structure [17]. Daynes et al. designed a pseudo periodic struts-based lattice structure whose cell size, aspect ratio, and orientation were changed following local principal stress [18]. Liu et al. realized functionally gradient lattice structures that smoothly vary along with stress intensity under the aid of Kriging interpolation [19].

Furthermore, the rationales of methods were integrated into conformal design. Hoang et al. proposed a multiscale topology optimization approach to directly map lattice materials for adaptive geometric components [20]. Wu et al. proposed a conformal gradient lattice design method in which lattice struts are generated along with both the contour of the object and principal stress [21]. Li et al. proposed an anisotropic gradient lattice design method under multiple physical fields (density field, directional field, gradient field) while the lattice structures are conformal to an arbitrarily 2.5D complex body [22]. Compared with topology optimization-based lattice infilling, stress field-driven methods are more flexible and detailed for populating lattice design.

Current research lacks effective evaluation and iteration for outputting results. These methods output local optimization rather than a global optimum. The main reason is that conventional finite element analysis (FEA) spends a prohibitive computational to evaluate GLMs. But considering the simple topology of strut-based lattice material, beam element analysis may be a good alternative [23]. It not only simplifies the analysis progress but makes a rapid evaluation of the lattice structure. This analysis method benefits an iterative optimization progress of the lattice structure.

However, considering lacking a systematic strategy for the design, evaluation, and optimization of lattice structures, this paper explores an integrative design method for conformal lattice structures for high stiffness. A sphere packing algorithm driven by a Vons Mises stress field is introduced to formulate the topologies of lattice structures. A sphere packing algorithm refers to populating a pile of adjacent circles but not overlapping into a given space, approximating the conformal transformation. Driven by the Vons Mises stress field, the size of each circle varies with the stress intensity; the higher the stress intensity is, the denser circles are. The centers of circles determine the distribution of lattice nodes. In addition, the Voronoi pattern and Delaunay triangular diagrams build the linking relationship among nodes and generate a skeleton of lattice structures. On this basis, truss elements analysis can replace the conventional FEA method to accelerate the performance evaluation. The design progress is integrated into the Genetic algorithm (GA) for optimizing design parameters. Iso-surface modeling morphs a series of optimized lattice topologies into organic and smooths these strut-based lattices for better mechanical efficiency. The generated lattice structures are compared with uniform body-centered cubic (BCC) lattices to verify the design methodology's feasibility.

This paper is organized as follows: in Section 2, a conformal lattice design method is presented, including sphere packing driven by stress intensity, linking means of nodes, iso-surface modelling, truss element evaluation for lattice frames, and parameters optimization by a Genetic algorithm. Section 3 presents a study case optimizing Messerschmitt-Bolkow-Blohm (MBB) beam by the proposed method; the designed lattice structures are compared with regular BCC lattice structure by experiential validation; this is followed by the conclusion in Section 4.

## 2 Design method

This part mainly demonstrates the design method for conformal GLMs. The circle packing algorithm driven by the von Mises stress field populates lattice nodes following stress intensity. Different approaches, including Voronoi and Delaunay triangular patterns, construct the topologies of lattice structures for a high-stiffness design. All the linking wires are transferred into thickened struts by iso-surface modeling technology. Truss element analyses are used to evaluate mechanical performance; the design parameters are optimized by a Genetic algorithm.

### 2.1 Populating nodes via circle packing driven by von Mises field

The circle packing algorithm is often applied in conformal design. The challenge is packing circles following the stress field. For the complex components, von Mises stress is used to evaluate the stress distribution. A mapping relationship between von Mises stress intensity and the node density of lattice structures were established. One is to build a continuous and stable stress intensity field to control the spheres' sizes. Another is packing all the spheres within the given 2D space.

#### 2.1.1 The generation of stress field

Von Mises stress $\sigma_{s}$, defined in equation (1), is an equivalent stress based on shear strain energy, which can reflect stress intensity suffered by given components.

(1) $$\begin{array}{c} \sigma_{s}=\sqrt{\frac{{\left(a_{1}-a_{2}\right)}^{2}+{\left(a_{2}-a_{3}\right)}^{2}+{\left(a_{3}-a_{1}\right)}^{2}}{2}} \end{array}$$

**Fig. 3:**
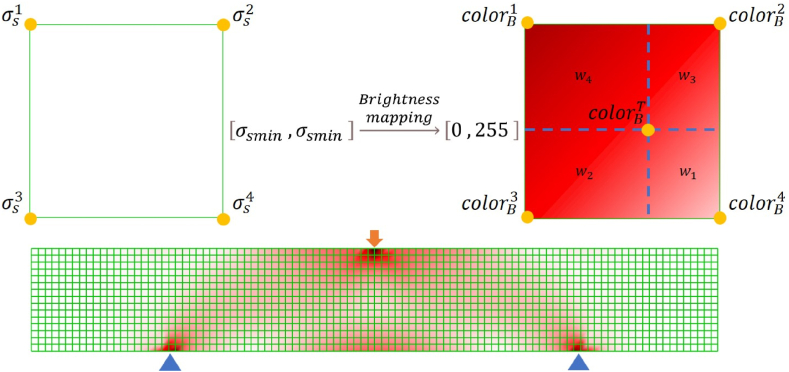
Mapping from stress field to color field. (For interpretation of the references to color in this figure legend, the reader is referred to the Web version of this article.)

Wherein, $a_{1},a_{2},a_{3}$ are denoted first, second and third principal stress, respectively. Geometric interpolations (i.e., interpolation of triangular barycentric coordinates) are applied for predicating von Mises stress at any position inside structures. For an intuitive expression, mesh rendering technologies store and indirectly illustrate stress intensity by color. The HSB model, describing color by H (Hue), S (Saturation), and B (Brightness), is utilized for transferring node stress efficiently to color. Brightness is set as a variable increasing as von Mises stress decreases, while Hue and Saturation are set as constants. All the nodes from FEA are employed to generate mesh, and only colored values at nodes need to render mesh, as seen in Fig. 3.

Bilinear interpolation is used to evaluate the ${color}_{B}^{T}$, shown in equation (2), by four known color values ${color}_{B}^{1}$, ${color}_{B}^{2}$, ${color}_{B}^{3}$, and ${color}_{B}^{4}$ .

(2) $$\begin{array}{c} \;{color}_{B}^{T}={w_{1}\times color}_{B}^{1}+{w_{2}\times color}_{B}^{2}+{w_{3}\times color}_{B}^{3}+{w_{4}\times color}_{B}^{4} \end{array}$$

$$w_{i}=\frac{{Area}_{w_{i}}}{{Area}_{total}}\;i=1,2,3,4$$

Where, $w_{i}$ is the corresponding weight value of each color value. It is determined by the rectangle area separated by the position of ${color}_{B}^{T}$.

To be noticed, von Mises stress often marked local stress concentration or evaluated possibly where industrial configurations destroyed, rather than a stress transmission. An exponential function enlarges the influence of the von Mises stress, as seen in equation (3).

(3) $$\begin{array}{c} G\left(x\right)=x^{n},n\in \left[1,n_{\max}\right] \end{array}$$

**Fig. 4:**
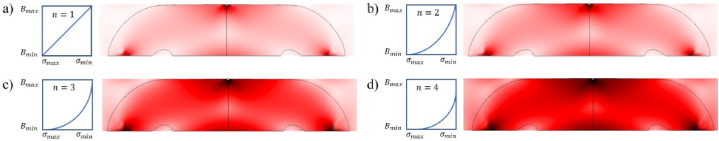
Mapping gradients a) n = 1 b) n = 2 c) n = 3 d) n = 4.

Where, n is power value used for expending the influence of von Mises stress and G(x) is a mapping function of color. The mapping results of different exponent values (n = 1, 2, 3, 4) are illustrated in Fig. 4 (a - d).

#### 2.1.2 Sphere packing driven by stress intensity

A circle packing algorithm refers to generating a pile of disks without overlapping but adjacent to each other [24]. This study considers the centers of spheres as lattice nodes to construct struts. A developed circle packing method is employed to populate lattice joints under the stress intensity field, expressed by HSB color, defined in equation (4).

(4) $$\begin{array}{c} H=\left\{\left(i,j\right):i\in I,j\in I,j<i\right\}\;while\;r_{i}=f\left(S_{color}\left(x_{i},y_{i}\right)\right)\in \left(r_{\min},r_{\max}\right)\\ f\left(S_{color}\left(x_{i},y_{i}\right)\right)=\frac{\left(255-color\left(x_{i},y_{i}\right)\right)\times \left(r_{\min}-r_{\max}\right)}{255}+r_{\min} \end{array}$$

Wherein, I is a set of points, and i,j elements of I. The radius and coordinates of $i_{th}$ point is $r_{i}$, and $\left(x_{i},y_{i}\right)$ , respectively. $r_{i}$ is determined by the position $\left(x_{i},y_{i}\right)$ of $i_{th}$ point in a color field $S_{color}$. $r_{\min}$ and $r_{\max}$ are the possible minimum and maximum radius of spheres, respectively. All the points $\left(x_{i},y_{i}\right)$ are restricted in the arbitrary 2D design domain Ω. At initial stage, all the points are randomly distributed in the Ω object.

The algorithm is based on Kangroo2, a plugin in Rhino-Grasshopper. The component, ImageCircles, is utilized for constructing a pile of disks in the design domain. The optimization problem can be summarized as equation (5).

(5) $$\begin{array}{c} \mathrm{Min}\left(U\left(X\right)\right)\\ s.t.\;\sqrt{{\left(x_{i}-x_{j}\right)}^{2}+{\left(y_{i}-y_{j}\right)}^{2}}\leq r_{i}+r_{j},\forall \left(i,j\right)\in H\\ r_{\min}<r_{i}<r_{\max},\left(x_{i},y_{i}\right)\in \Omega \forall i\in H \end{array}$$

Here, $r_{i}$ is a known real positive number, while $r_{i}$ and $r_{j}$ are the radius of $i_{th}$ and $j_{th}$ disks, respectively.

(6) $$\begin{array}{c} \vec{F_{i}}=\vec{f_{1}}+\vec{f_{2}}+,\ldots ,+\vec{f_{k}},k=1,\ldots ,n \end{array}$$

Assume the positions of these objects are known. There is mutual embedding between the i,th and the $j_{th}$ objects. By loosely applying Hook's law without affecting the validity of the results in terms of packing, the $\vec{F_{i}}$ in equation (6) is considered as proportional to the embedding depths. The directions of these forces are all toward the centers of the disks. Herein, equation (7) defines the magnitude of the moving vector, and equation (8) defines the moving direction.

(7) $$\begin{array}{c} \left|\vec{f_{k}}\right|=\left|\sqrt{{\left(x_{i}-x_{j}\right)}^{2}+{\left(y_{i}-y_{j}\right)}^{2}}-\left(r_{i}+r_{j}\right)\right| \end{array}$$

(8) $$\begin{array}{c} \frac{\vec{f_{k}}}{\left|\vec{f_{k}}\right|}=\frac{\left(x_{i}+x_{j}\right)\vec{e_{1}}+\left(y_{i}+y_{j}\right)\vec{e_{2}}}{\left|\left(x_{i}+x_{j}\right)\vec{e_{1}}+\left(y_{i}+y_{j}\right)\vec{e_{2}}\right|} \end{array}$$

Where, $\vec{e_{1}}$ and $\vec{e_{2}}$ are unit vectors along the x and y axes, respectively, in the Cartesian system. Within a small time interval, the movement of the i th bin is a small step along the total force, defined in equation (9).

(9) $$\begin{array}{c} \left(x_{i}^{t+1}\vec{e_{1}}+y_{i}^{t+1}\vec{e_{2}}\right)=\left(x_{i}^{t}\vec{e_{1}}+y_{i}^{t}\vec{e_{2}}\right)+\varepsilon \vec{F_{i}} \end{array}$$

ε is a small positive constant. The consequence of this small movement is a reduction of mutual squeezing. The surface is dispersed by those disks.

(10) $$\begin{array}{c} U\left(X\right)=U\left(x_{1},y_{1},x_{2},y_{2},\ldots ,x_{n},y_{n}\right)=\sum_{j=1}^{n}U_{j} \end{array}$$

(11) $$\begin{array}{c} U_{j}=\sum_{i=1,j\neq 1}^{n}U_{ij},j=1,2,\ldots ,n \end{array}$$

(12) $$\begin{array}{c} u_{ij}=kd_{ij}^{2},i,j=1,\ldots ,n\;i\neq j \end{array}$$

**Fig. 5:**
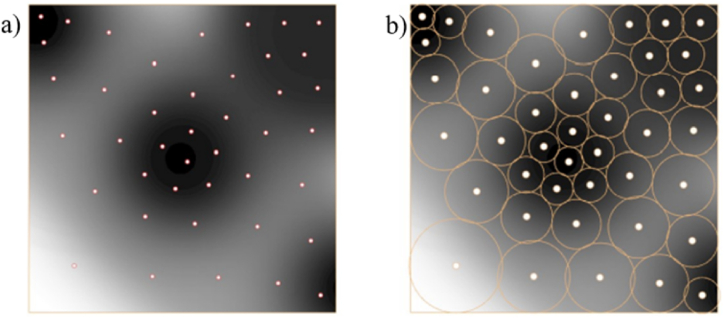
Circle packing algorithm a) nodes from circles b) a pile of circles.

Similarly, in equation 10–12, $u_{ij}$ and $d_{ij}$ are the squeezing potential and the embedding depth between the ith and the jth points, respectively. $U_{j}$ is total squeezing potential of $j_{th}$ disk. U(X) is a collective squeezing potential of all disks. Circle packing finishes when the total potential of all disks U(X) converged to a given minimum, as shown in Fig. 5 (a, b).

### 2.2 Exploring the frames of struts-based lattice structure

**Fig. 6:**
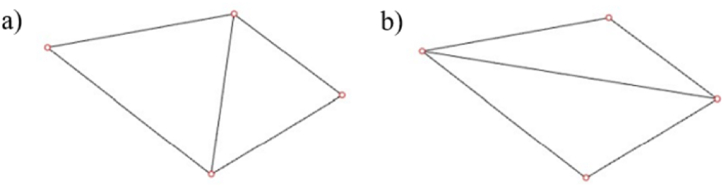
Triangulation a) Delaunay triangulation b) Other methods.

Two approaches, including Delaunay triangular and the Voronoi polygon, are used to define lattice topologies. Delaunay triangulation of point set P on a plane is a triangulation DT (P), so that no point in P is strictly located inside the circumscribed circle of any triangle in DT (P). This algorithm can connect the points on the plane into stable triangles, which means it will maximize the internal angles of all the generated triangles and try to avoid “skinny” triangles, as shown in Fig. 6 (a, b).

**Fig. 7:**
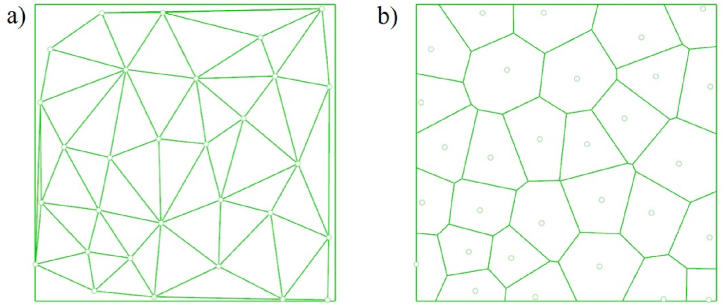
Different linking methods a) Delaunay trianglar b) Voronoi polygon.

Considering the structural efficiency, triangles constructed by this algorithm are approximated to equilateral triangles, the mechanical properties of which are very stable, as shown in Fig. 7(a).

A Voronoi diagram is a group of polygons next to each other; each polygon is composed of vertical bisectors between two adjacent points, as seen in Fig. 7 (b). Amounts of different points on the plane are divided according to the nearest neighbor principle; Each point is associated with its nearest neighbor region. For $P_{k}$ in a point set {$P_{0}$, $P_{1}$,…, $P_{n}$}, its Voronoi region $R_{k}$ is defined in equation (13).

(13) $$\begin{array}{c} R_{k}=\left\{x\in R^{3}|d\left(x,P_{k}\right)<d\left(x,P_{j}\right),j=\left\{0,1,2,\ldots ,n\right\},j\neq k\right\} \end{array}$$

**Fig. 8:**
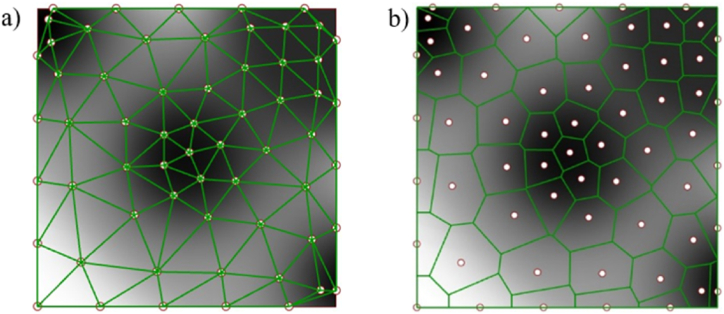
Gradient linking methods a) Gradient Delaunay triangular b) Gradient Voronoi polygon.

It is worth noticing that the Delaunay triangle is a triangle formed by connecting related points that share an edge with adjacent Voronoi polygons, as illustrated in Fig. 8 (a, b).

Ashby's research has elaborated that the polygon of Voronoi results in bending-dominated mechanical behavior, and Delaunay triangular has stretched-dominated mechanical behavior [25]. Both linking methods are taken for a comparison study.

### 2.3 Constructing organic morphology by struts-based lattice frames

Generally, boundary representation(B-rep) and constructive solid geometry (CSG) often model the struts-based model in the commercial software [26]. However, they are not suitable for complex, intricate lattices, especially quasi-periodic lattices, owing to prohibitive computational costs [27]. Iso-surface is a good substitute. It is a 3D surface where all points present the same weight values [28]. Lattice wireframes can be warped into thickened struts by this modelling method.

**Fig. 9:**
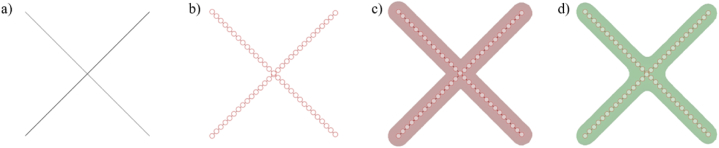
The generation of organic lattice a) lattice topology b) discrete points pattern c) thicken struts by iso-surface modelling d) smoothing organic lattice.

Herein, Dendro (ECR LABS, Los Angeles, USA), an open-source plugin for Grasshopper™ in the Rhinoceros software package ® (Robert McNeel & Associates, Seattle, USA), is utilized for modeling and smoothing lattice. The plugin is designed to work on dense point clouds for a voxel-based iso-surface. A line wireframe is divided into points pattern, and then an iso surface modeling is built from points. Fig. 9 (a - d) presents the generation process of an organic porous structure.

**Fig. 10:**
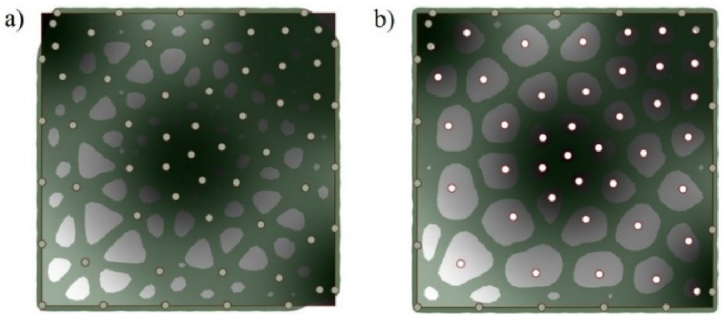
Organic structure by iso-surface modelling a) Organic Delaunay triangular lattice structure b) Organic Voronoi lattice structure.

In addition, based on dense varying lattice frames, the solid-lattice hybrid lattice structure lattice structure is generated, as seen in Fig. 10.

### 2.4 Parametric optimization and performance evaluation

After the design phase, the mechanical performance of the designed lattice structures is required for evaluation, and the design parameters, including Node number, mapping gradient, and the range of varying circle size, greatly influence the generated lattice structure. A structural performance feedback loop is established for parameter optimization by the Genetic algorithm, described by Algorithm 1.

**Table:** 

**Algorithm 1** Pseudocode of GA Framework
1: population ←generate 200 parametric combinations 2: **for** generation_number = 1 → 100 **do** 3: evaluate all parametric combinations by truss analysis to mark the fitness score 4: new_population ← NULL 5: copy 15% best parametric combinations to new_population 6: **while** size (new_population) ≤ 200 **do** 7: parent1 ← select parametric combinations 8: parent2 ← select parametric combinations 9: child ←*crossover* (parent1, parent2) 10: add child to new_population 11: **end while** 12: population← new_population 13: **end for**

**Fig. 11:**
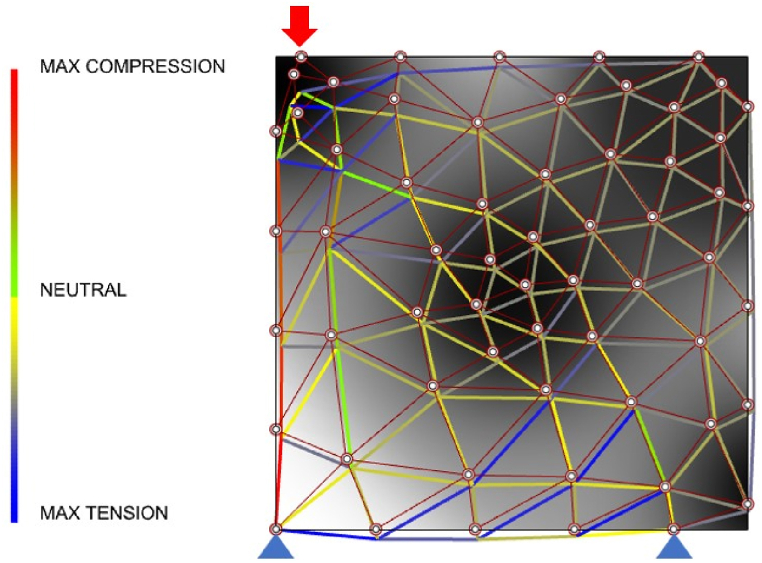
Beam elements analysis.

In the loop, beam analysis is a simplified and efficient alternative model to evaluate lattice structure, and a Genetic algorithm outputs a series of feasible solutions. The Link180 element handles the stress and deformation of lattice structures. In an example of Fig. 11, a reference load is imposed on the top of the lattice structure, and the bottom is fixed.

In the quantitative analysis and by the elimination of inferior parameter combinations, the tactic obtains for the relatively satisfactory solutions by Genetic algorithm. The optimization problem can be defined as minimizing the max displacement δ of lattice structures under the same loads, as shown in equation (14).

(14) $$\begin{array}{c} \min\;\delta \left(G,P,r_{\min},r_{\max}\right)\\ s.t.\;G\left(x_{1}\right),x_{1}\in \left[1.0,G_{\max}\right]\\ r_{\min}\in \left[r_{a},r_{b}\right];r_{\max}\in \left[r_{b},r_{c}\right]\\ P\in \left[N_{\min},N_{\max}\right]\\ V\left(x_{3}\right)=V^{0}\\ \left(x_{i},y_{i}\right)\in \Omega ,i=1,2,3,\ldots ,P \end{array}$$

P is the number of lattice nodes. $G\left(x_{1}\right)$ is the mapping gradient to expend the effect of von Mises stress, as described in section 2.1.1. $G_{\max}$ is maximum exponent value. The varying range $\left[N_{\min},N_{\max}\right]$ can be set as a rough scope. And all the points ($x_{i},y_{i})$ are restricted in a given design domain Ω. $r_{\min}$ and $r_{\max}$ are the varying size of circles. Their optimized ranges are determined by $r_{a},r_{b}$, and $r_{c}$. $V^{0}$ is a given total volume of the lattice truss. $r_{a}$ refers to the low limitation of $r_{\min}$, determined by fabrication accuracy; $r_{c}$, defined in equation (15), refers to the up limitation of $r_{\max}$; $r_{b}$ is an intermediate value between $r_{a}$ and $r_{c}$. It is a rough estimated value which is relate to the area of infilling target and the number of nodes.

(15) $$\begin{array}{c} r_{c}=\sqrt{\frac{A}{N_{\min}\pi }} \end{array}$$

Where, $N_{\min}$ is the lower boundary of the number of points, A is the area of design domain. A series of feasible lattice topologies can be obtained after genetic algorithm converged, as shown in Algorithm 2:

**Table:** 

**Algorithm 2** Filtering Parametric combination in the converging population
1: *population* ← generate 200 parametric combinations 2: four sets of each kind variables ← *[*$r_{\min}$*,*$r_{\max}$*,*P*,*G*]* 3: count the repeated times of parameters in each set 4: get the parameter density distribution of every kind of variables 5: sample parameters with high density, then re-combine each kind variable

The filtered parameters can be filtered and used to generate an organic lattice structure.

## 3 Experimental validation and discussion

A two-dimensional (2D) bending beam is utilized for numerical simulation to validate the proposed method. Then 2.5-dimensional (2.5D) numerical examples are provided to further illustrate the effectiveness of the proposed method through physical experiments.

### 3.1 Design for 2D MBB beam

**Fig. 12:**
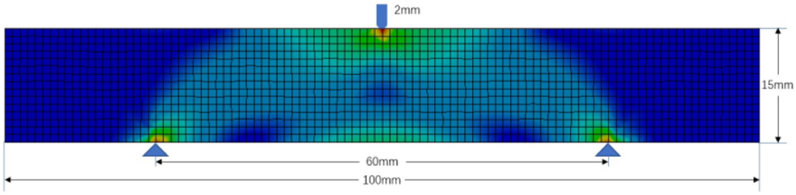
Finite element analysis for 2D MMB beam.

As shown in Fig. 12, a two-dimensional MBB beam with a length of 100 mm, a height of 15 mm, and a thickness of 1 mm is fixed on two support pins spaced 60 mm apart. A displacement of 2 mm is imposed at the top center point of the beam edge. One thousand five hundred quad elements discretize this 2D beam. The material is set as *FormLab3* SLA white resin, and its material property is illustrated in Table 1. It is found that the maximum equivalent stress is 35.957 MPa located at the loading position, less than the yield strength of 38.303 MPa, indicating the structure in the elastic deformation.

**Table 1:** Material properties of FormLab3 white resin.

Density	Young's modulus	Poisson's Ratio	Yield Strength
1100 $kg/m^{3}$	251.4 MPa	0.23	38.303 MPa

**Fig. 13:**
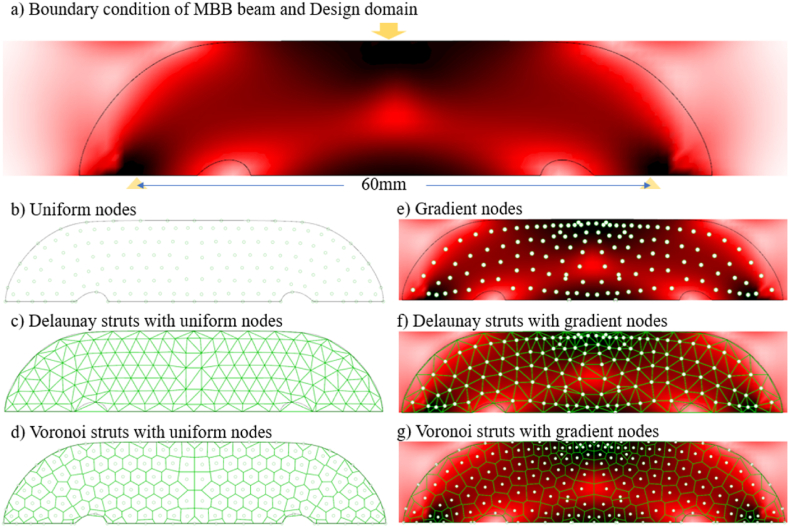
Comparisons between 2D MBB beams.

Meanwhile, an irregular design domain is defined, as seen in Fig. 13 (a). The domain with high von Mises stress is employed in infilling strut-based lattices, while the others can be removed. The 72 nodes are located within the design domain. The beam elements analysis method is used to verify the structural efficiency of the strut-based lattice structure, and the global volume constraint is controlled to around 25 ${mm}^{3}$. The uniform nodes are populated as Fig. 13 (b - d), whereas the optimized designs of gradient lattice for the 2D MBB beam are shown in Fig. 13 (e − g). Truss beam element analysis can efficiently evaluate the mechanical properties of the designed structures. The gradient strut-based lattice structure produces around 1.56 mm deformation smaller than 1.70 mm of uniform strut-based lattice under the 20 N load. It implies that gradient strut-based lattice shows preferable stiffness in the two-dimensional design domain.

### 3.2 Infilling lattice to 2.5D MBB beam

**Fig. 14:**
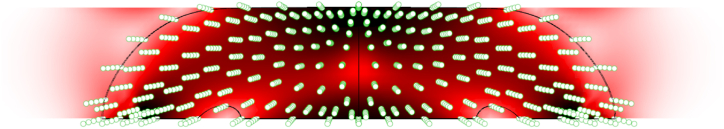
Extruding nodes into 2.5D space.

The proposed method generates conformal and gradient structures from the 2D stress field. Extruding nodes into 3D space is essential for arbitrary 2.5D components, as shown in Fig. 14.

To guarantee numerical evaluation near the practical industry scenario, three-point bending experiment is used to test all the beams to verify the feasibility of the proposed methods. The experiment set follows GB/T 1456–2005 standard. A bending beam with 100 mm × 10 mm × 15 mm (L × W × H) is taken as a case model. The nodes generated in two-dimensional plane are replicated and moved into 2.5-dimensional space, as shown in Fig. 14. The span of the beam is 60 mm, as shown in Fig. 14. The gradient lattice structures by the proposed method are going to compare with conformal uniform BCC lattice structures. Bending stiffness and max bearing capacity are considered as two main comparison criteria. For a 2.5-dimensional study case, a finite element analysis is carried out under the external force 20 N, which ensures the elastic deformation of the beam structure. The material properties of the bending beam are set as the same in section 3.1.

#### 3.2.1 Combinational optimization by genetic algorithm

The problem of parameter optimization is set as described in equation (16).

(16) $$\begin{array}{c} \min\;\delta \left(G,P,r_{\min},r_{\max}\right)\\ s.t.\;G\left(x_{1}\right),x_{1}\in \left[1.0,10.0\right]\\ P\in \left[25,100\right]\\ r_{\min}\in \left[0.5,1.5\right];r_{\max}\in \left[1.5,3.5\right]\\ V\left(x_{3}\right)=3800\;{mm}^{3}\\ \left(x_{i},y_{i}\right)\in \Omega ,i=1,2,3,\ldots ,P \end{array}$$

**Fig. 15:**
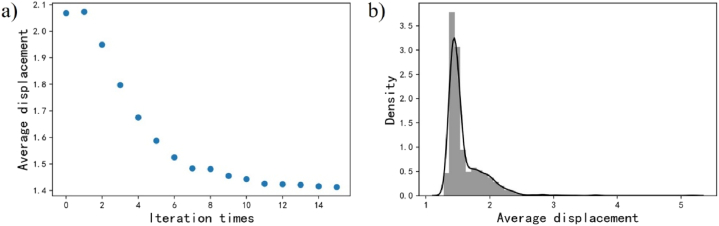
Optimization by Genetic algorithm a) An efficient converging progress b) Converging range in the 15th generation.

Where, Ω is the same as the region in Fig. 13 (a); its area is 906 ${mm}^{2}$. The point number P varies from 25 to 100. $r_{\min}$ and $r_{\max}$ change in ranges [0.5, 1.5] and [1.5,3.5], respectively. The upper boundary of $r_{\max}$ is defined by equation (15). The volume of all the samples is restricted in $3800\;{mm}^{3}$. Genetic algorithm solves the current problem. Each population has 200 individuals. The results start to converge in the 12th iteration and optimizing results stabilize in the 15th iteration, as seen in Fig. 15 (a, b).

**Figure 16:**
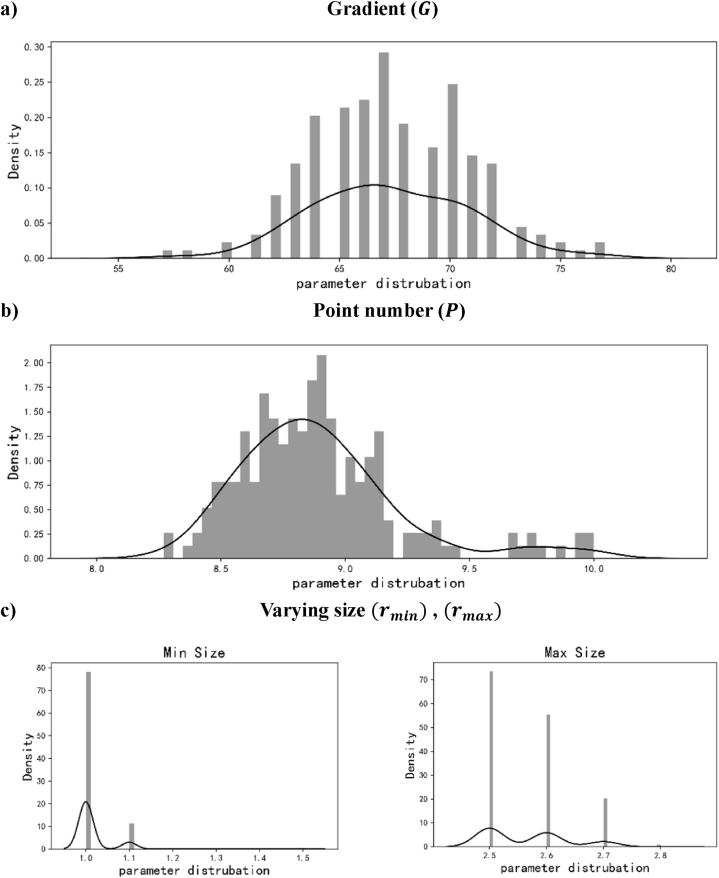
Parameter distribution by Genetic algorithm a) Gradient b) Point number c) Varying size

Hence, a quantitative analysis is conducted for the 15th iteration so as to seek to a feasible series of parameter combinations. The density distribution of design parameters is illustrated, as shown in Fig. 16. Based on the density distribution of all the parameters, the parameters with high occurring frequency were combined and inputted for constructing lattice structures.

In Fig. 16 (c), it is found that $r_{\min}$ and $r_{\max}$ are narrowed to specific ranges, [1.0,1.1] and [2.5, 2.6, 2.7], respectively. The distribution of G and P are relatively discrete, as seen in Fig. 16 (a, b). Hence, the mode numbers of G and P are selected and then make a permutation for the that $r_{\min}$ and $r_{\max}$. The six parameter combinations can be used for generating high-stiffness porous structure, as shown in Table 2.

**Table 2:** Parameter combinations.

ID	Gradient	Nodes	Minimize size	Maximize size
CP1	8.8	66	1.0	2.5
CP2			1.0	2.6
CP3			1.0	2.7
CP4			1.1	2.5
CP5			1.1	2.6
CP6			1.1	2.7

#### 3.2.2 The comparison of mechanical performance

The mechanical performance of the generated structure will be used for comparison with conformal BCC structures. All the samples were manufactured by SLA technology via FormLab3; this device manufactures all samples accurately and efficiently without more redundant supporting structures. Its fabricating parameters and material properties are shown in Table 3.

**Table 3:** Fabricating and Post processing parameters.

Fabricating parameters	Heat platform temperature (min)	39
	Layer thickness (mm)	0.1
	Min feature size	0.5
Post processing parameters	Washing by isopropyl alcohol (min)	10
	Baking (°C−min)	60–60

**Fig. 17:**
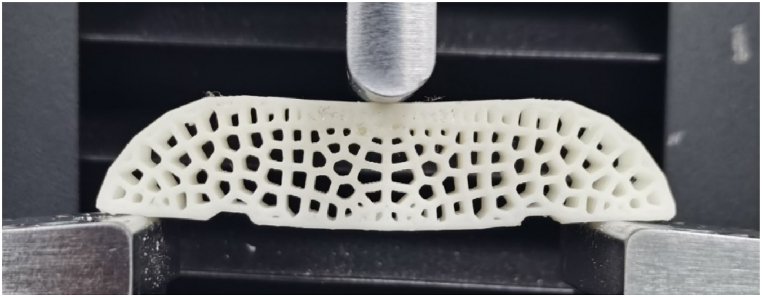
Experimental setup.

**Fig. 18:**
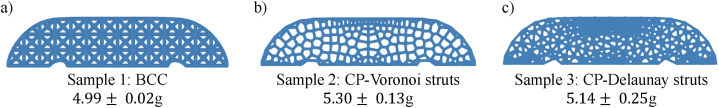
Testing samples a) BCC struts b) CP-Voronoi struts c) CP-Delaunay struts.

The samples are tested by the Sansizonghe UTM2000 testing machine, as shown in Fig. 17. The displacement of 1 mm/min is loaded on all the samples. The volumes of all samples are reduced to 25% of the original solid beams. Conformal BCC lattice is regarded as the control group to verify the structural efficiency of the designed lattice structure. The mass of the samples is controlled at around 5.00 g, as shown in Fig. 18.

A Force-Displacement curve is obtained by three points bending experiments. According to the results of experiments, the bending stiffnesses values of all simples are calculated with following equation (17):

(17) $$\begin{array}{c} E_{b}=\frac{L^{3}}{48I_{z}}\times \left(\frac{\Delta P}{\Delta F}\right) \end{array}$$

Where, $E_{b}$ is denoted by the bending stiffness. L is the span between support points. $I_{z}$ is the moment of inertia of the section of each sample. P is a load suffered by samples, and F is a displacement. ΔP/ΔF is the slope of the force-displacement curve when the linear elastic deformation happens. Herein, when the deformation is small, in order to simplify the analysis, the sensor displacement is approximately taken as the deflection F of the sample. Each structure produced five samples for comparison.

**Fig. 19:**
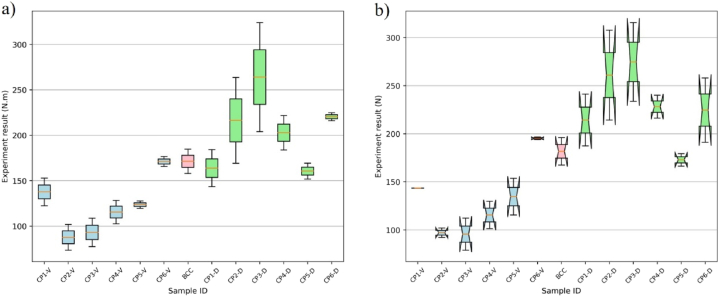
Mechanical performance for different samples a) Bending stiffness b) Max bearing force.

As seen in Fig. 19 (a, b), except CP1 and CP5, the stiffness of the Delaunay-strut structures has a remarkable increase over that of conformal BCC lattice structure, improved to 1.86 times at most. Similarly, the maximum bearing capacity of the Delaunay-strut structures also is increased to 1.5 times that of the conformal BCC lattice structure. By comparison, the Voronoi lattice structure did not show better stiffness and bearing capacity. The reason is that Voronoi structure has bending-dominated mechanical behavior, not adapting to high stiffness design.

Under the proposed method, the dense varied of the proposed lattice follow stress intensity. The higher the stress intensity is, the denser the strut-based lattices are, as seen in Fig. 18 (c). In a high-stress region, the designed lattice structures have a high relative elastic modulus to efficiently resist external loads during elastic deformation compared to regular BCC lattice structures. On the material level, Delaunay struts model is stretch-dominated and has an organic morphology. Therefore, the designed gradient strut-based lattice cluster presents preferable bending stiffness against regular BCC lattice structures.

## 4 Conclusion

This paper presents an integrative method to design and optimize conformal struts-based lattice structures. The sphere packing algorithm driven by stress field populates the lattice nodes efficiently in an arbitrary 2.5D design domain, and the size of the sphere is regulated by stress intensity expressed by the HSB color model. All nodes are linked by different linking means, including the Delaunay diagram and the Voronoi diagram. The iso-surface modeling technology thickens and smooths all linking lines into solid struts to improve the elastic modulus of lattice material. Besides the lattice design, a beam element analysis evaluates the structural efficiency of a lattice structure, while a Genetic algorithm optimizes design parameters.

Via the three-point bending experiments, it is found that the proposed Dulaney lattices present superb stiffness among all design samples. Specifically, the Dulaney lattice has preferable mechanical performance in stiffness and strength compared with the Voronoi scheme and conformal BCC lattice structure.

Furthermore, the method is proved suitable for the lightweight design of any arbitrary 2.5D components under the support of additive manufacturing. On the one hand, design outputs can reserve the geometric characteristic of 2.5D components. On the other, the sphere packing algorithm may efficiently restrict the distance between nodes so that the pore size can be effectively controlled for different additively fabricating schemes. The research delivers a good reference for lattice-based conformal optimization of complex 2.5D geometric components in aviation, aerospace, and other industrial fields.

## Author contribution statement

Fuyuan Liu: Conceived and designed the experiments; Performed the experiments; Analyzed and interpreted the data; Wrote the paper.

Min Chen, Lizhe Wang, Tianheng Luo: Performed the experiments; Analyzed and interpreted the data.

Geng Chen: Contributed reagents, materials, analysis tools or data.

## Funding statement

Mr. Fuyuan Liu was supported by National International Science and Technology Cooperation Base on Railway Vehicle Operation Engineering of Beijing Jiaotong University [BMRV21KF07 & BMRV20KF03], XJTLU Research Development Fund [RDF-17-02-44 & RDF-SP-122].

This work was supported by National Natural Science Foundation of China [52075033].

## Data availability statement

Data associated with this study has been deposited at DOI:10.17632/8crtg79jt7.1.

## Declaration of interest's statement

The authors declare no competing interests.
